# Rising of pyrethroid resistance mutations (kdr) in the dengue vector *Aedes aegypti* from northeastern Argentina

**DOI:** 10.3389/fpubh.2026.1754455

**Published:** 2026-05-20

**Authors:** Jessica V. Fay, Sonia L. Espindola, Belen Gallara, Rodrigo Zarate, Fabian Zelaya, Solange M. Busch, Maria J. Blariza, Carina F. Argüelles, Julian A. Ferreras, Marcos M. Miretti

**Affiliations:** 1 Laboratorio GIGA, Instituto de Biología Subtropical, FCEQyN, Universidad Nacional de Misiones - Consejo Nacional de Investigaciones Científicas y Técnicas, Posadas, Misiones, Argentina; 2 Centro Misionero de Investigación y Control de Enfermedades Vectoriales y Zoonóticas, Dirección de Saneamiento Ambiental, Ministerio de Salud Pública de la Provincia de Misiones, Posadas, Argentina

**Keywords:** *Aedes aegypti*, arboviral vector, insecticide resistance, knockdown resistance (kdr), pyrethroid resistance, V1016I F1534C mutations, vector control, voltage-gated sodium channel

## Abstract

Insecticide resistance poses a significant threat to the control of the main dengue vector, *Aedes aegypti*. Knockdown resistance mutations (kdr) have been associated with pyrethroid resistance and embody the primary source of genetically resistant mosquitoes. This study describes the evolution of two kdr mutations, V1016I and F1534C, in *Ae. aegypti* from Posadas, Argentina, based on TaqMan genotyping data generated from two sampling times, 2019 and 2024, at the same collection sites. Consistent with global studies, we observed a significant increase in the frequency of these kdr alleles (>14%) after 5 years, where 93% of samples had at least two kdr alleles. Most remarkably, the number of highly resistant mosquitoes carrying three or four kdr alleles have expanded 25% in 5 years. The rapid rise in pyrethroid genetic resistance might result from the intense insecticide use during dengue outbreaks. This 5% yearly rate trend highlights the need to integrate routine genetic resistance monitoring into vector control programs, and to adopt alternative strategies to prevent further erosion of insecticides efficacy.

## Introduction

*Aedes aegypti* currently stands as the most critical arboviral diseases vector worldwide (1, 2), while *Aedes albopictus* plays a secondary role in regional transmission dynamics in the Americas (3, 4). South America has experienced an alarming increase in dengue incidence in recent years, reaching a global record of dengue infections in 2024 with 13 million of reported cases (5). This scenario has been further worsened by the spread of the Zika and chikungunya viruses (6, 7). Argentina ranked second among countries with the highest records of dengue infections (581,559 cases), though reporting a lower number of chikungunya (1,388) and Zika (564) cases (5).

Pyrethroid-based insecticides remain the most widely used vector control tool owing to their rapid knockdown effect, high efficacy and relative safety for non-target species (8, 9). However, their effectiveness has been increasingly undermined by the rapid development of resistance in *Ae. aegypti* populations (10–12). There are several mechanisms involved in insecticide resistance, knockdown resistance (kdr) mutations constitute the primary cause of pyrethroid insecticide resistance documented to date (12–14). These mutations introduce amino acid substitutions in the neuron voltage-gated sodium channel protein (VGSC) reducing pyrethroid binding affinity and enabling mosquitoes to survive otherwise lethal exposures (15–18).

The valine-to-isoleucine substitutions at position p.1016 of the VGSC protein (V1016I) and the phenylalanine-to-cysteine substitution at position p.1534 (F1534C) are the most frequent kdr mutations described worldwide with frequencies exceeding 90% in some South American countries (8). Their co-occurrence has been associated with a greater reduction in VGSC sensitivity, therefore enhancing pyrethroid resistance (1, 9, 11, 15, 16, 19–22). While the F1534C and V1016I mutations are prevalent in highly resistant *Ae. aegypti* populations (23), other mutations have also been related to pyrethroid resistance in specific geographic regions, e.g., V410L, L982W, S989P, I1011M, V1016G (8, 24).

Several studies strongly correlate presence and dosage of kdr alleles with phenotypic resistance, reinforcing the essential role of these mutations in mediating pyrethroid resistance (14, 25). The presence of one or two copies of the IC-resistant haplotype, i.e., p.1016I/p.1534C, conferred a 20- to 60-fold increase in pyrethroid resistance (26). Fan and Scott (14) have also demonstrated a 7- to 16-fold increase associated with the 1534C allele. Kdr genotyping has therefore emerged as a reliable, rapid and scalable predictor of pyrethroid resistance (25, 26).

In a previous study, we reported the presence of the V1016I and F1534C mutations in adult *Ae. aegypti* collected in 2019 in Posadas, Argentina, and documented heterogeneity in genotype frequencies across neighbourhoods (27). Subsequent studies have also identified kdr mutations, including V410L, in other Argentinian cities (28–30), yet no long-term assessments have evaluated temporal changes in insecticide resistance-associated alleles in the country.

A rapid increase of the V1016I and F1534C kdr frequencies have been documented worldwide, particularly in Mexico, Brazil, Colombia and Peru (21, 31–34). In this report we assess pyrethroid genetic resistance dynamics in *Ae. aegypti* from Posadas after 5 years and following two major dengue epidemies.

## Methods

### Study site and biological material

To determine the temporal variation in kdr frequencies, the sampling was carried out in the same Posadas neighbourhoods investigated in 2019, namely Villa Sarita (VS), Palomar (BP), San Lorenzo (SL), and Nueva Esperanza (NE), where kdr mutations were first reported in local *Ae. aegypti* populations (27). *Ae. aegypti* eggs were collected using ovitraps (black plastic and paper jars) placed intra- and peri-domiciliary in households across the studied neighbourhoods between February and May 2024. Ovitraps were placed at the same time in all neighbourhoods, being replaced with new ones every 7 days in three occasions. Mosquitoes’ eggs were transported to the laboratory, reared to adulthood and fed a 10% sugar solution. Adult mosquitoes were cryopreserved until DNA extraction.

### Identification of knockdown resistance mutations

DNA was obtained from 129 adult *Ae. aegypti* mosquitoes (F0) derived from 4 neighbourhoods in Posadas (VS = 33, BP = 32, SL = 33, and NE = 31) using a commercial kit (Wizard Genomic DNA Purification Kit, Promega). Direct genotyping of V1016I and F1534C kdr mutations in the *Nav* gene was performed using custom TaqMan SNP genotyping assays (ThermoFisher) as described in Melo Costa et al. (10) using individually extracted DNA as template. A non-template reaction was included as a negative control and DNA from previously confirmed resistant genotypes was used as positive controls. Primer and probe sequences, as well as reaction conditions, are provided in Supplementary Table 1.

Allelic and genotypic frequencies were calculated by direct counting at each locus (*Nav* p.1016: VV, VI, II; *Nav p.*1534: FF, FC, CC). Combined genotypes (V1016I + F1534C), were also estimated (i.e., VV/FF, VV/FC, VV/CC, VI/FF, VI/FC, VI/CC, II/FF, II/FC, II/CC). The Z-score was used to evaluate allelic and genotypic frequencies changes between two sampling times, 2019 vs. 2024.

## Results

Genotyping of the V1016I and F1534C kdr mutations in 129 mosquitoes collected in 2024 in Posadas, Argentina, revealed that 99% of individuals carried at least one resistance-associated allele, and 93% carried two kdr alleles. Estimated allelic frequencies of the resistance variants were 44.57% for 1016I and 83.72% for 1534C (Table 1).

**Table 1:** Frequency of knockdown resistance (kdr) alleles and genotypes found at *Nav* p.1016 and *Nav* p.1534 in *Aedes aegypti* from Posadas, Argentina.

KDR position	Allele	Allelic frequency %		Genotype	**2019**		**2024**	
		**2019**	**2024**		*n*	Frequency ^a^ (%)	*n*	Frequency ^a^ (%)
**1016**	**V**	70.91	55.42	**VV**	50	51.02	29	22.48
				**VI**	39	39.80	85	65.89
	I*	29.08**	44.57**	**II**	9	9.18	15	11.63
					**98**		**129**	
**1534**	**F**	29.29	16.27	**FF**	8	8.08	1	0.78
				**FC**	42	42.42	40	31.00
	C*	70.70**	83.72**	**CC**	49	49.49	88	68.22
					**99**		**129**	

*Knockdown alleles at Nav p.1016 and Nav p.1534. **Significant differences in kdr allele frequencies in 2019 vs. 2024 (*p* < 0,001). ^a^Frequency represents relative frequencies expressed as percentage. Bold values in columns “*n*” indicate total number of mosquitoes evaluated.

All genotypes were detected (*Nav* p.1016: VV, VI, II; *Nav* p.1534: FF, FC, CC). Heterozygotes at position p.1016 (VI = 65.89%) were the most common, while at position p.1534 the resistant homozygotes (CC = 68.22%) prevailed (Table 1).

Genotype frequencies met Hardy–Weinberg expectations at *Nav* p.1534 but not at *Nav* p. 1016, where a significant excess of heterozygote was detected. When considering combined genotypes, VI/CC was the most frequent (43.41%). The fully susceptible double homozygote (VV/FF) was rare 0.77% while the double resistance homozygote (II/CC) reached 9.30% (Table 2).

**Table 2:** Frequency (%) of combined kdr genotypes (V1016I + F1534C) in *Aedes aegypti* from Posadas, Argentina.

Combined genotype*	**2019**		**2024**	
	*n*	Frequency^**a**^ (%)	*n*	Frequency^**a**^ (%)
VV/FF (SS/SS)	7	7.37	1	0.77
VV/FC (SSS**R**)	22	23.16	8	6.20
VV/CC (SS/**RR**)	19	20.00	20	15.50
VI/FC (S**R/**S**R**)	19	20.00	29	22.48
VI/CC (S**R/RR**)**	20	21.05	56	43.41
II/FC (**RR/**S**R**)**	0	0.00	3	2.32
II/CC (**RR/RR**)**	8	8.42	12	9.30
	**95**		**129**	

S, susceptible allele; **R**, resistant allele. *VI/FF, IIFF absent genotypes. **significant differences in the total number of highly resistant mosquitoes carrying three or four resistant alleles between 2019 and 2024 (*p* < 0,001). ^a^frequency represents relative frequencies expressed as percentage. Bold values in columns “*n*” indicate total number of mosquitoes evaluated.

**Figure 1:**
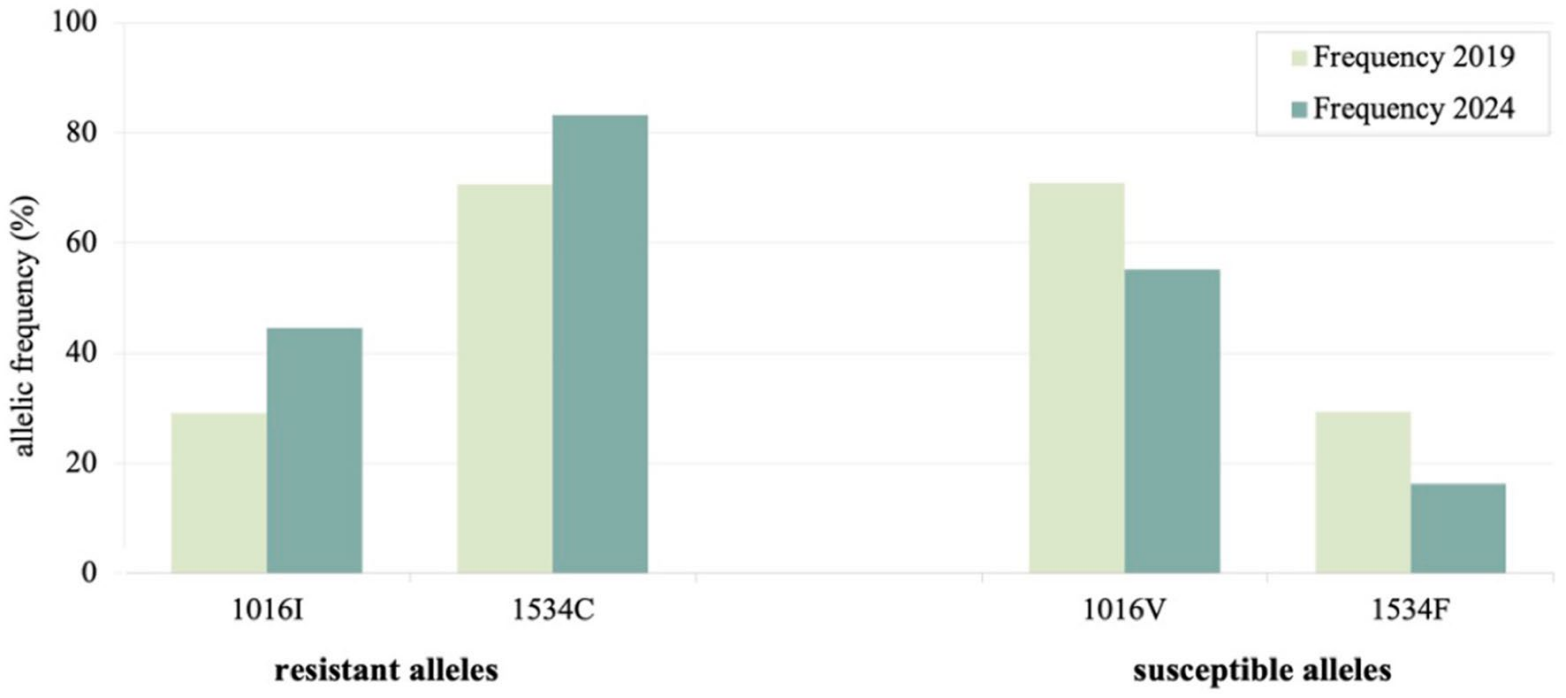
Changes in the resistant and susceptible allele frequencies (V1016I and F1534C) between *Aedes aegypti* sampled in 2019 and 2024 in Posadas, Argentina.

Substantial increases in resistant allele frequencies were observed relative to the 2019 (27), with kdr 1016I rising 15% and kdr 1534C 13% (*p* < 0.001, Table 1). Figure 1 shows the resistant and susceptible allelic frequencies shifts between 2019 and 2024.

Resistant homozygotes CC and II increased 18.72 and 2.44% respectively, while susceptible homozygotes markedly decreased (VV: 24.48%; FF: 0.78%) (Table 1). Combined genotypes considering both *Nav* sites, p.1016 and p.1534, showed that the proportion of mosquitoes carrying three or more resistant alleles increased 25.56% (*p* < 0.001), whereas those with three or more susceptible alleles declined 23.56% in 5 years (Table 2).

## Discussion

Insecticide resistance represents a worldwide concern in arboviral diseases control and has profound impact in public health. The intensive use of pyrethroid-based insecticides in recent dengue epidemics strengthened the selective process and further spread knockdown resistance alleles. Numerous studies have consistently demonstrated the central role of F1534C and V1016I kdr mutations on *Ae. aegypti* pyrethroid resistance (1, 14–16, 19–22, 25, 26). These reports reinforce the use of kdr genotyping as a strong predictor of pyrethroid resistant phenotypes. Reports in several Latin American countries reveal a noteworthy expansion in the prevalence of kdr mutations, e.g., 1016I and 1534C frequencies in Brazil considerably rose in 6 years (21, 34), and in Mexico 1016I increased from 0.1 to 88.3% over 7–9 years (33). Although kdr mutations have been detected in northern and central Argentina (27–30), temporal trends on kdr allelic frequencies have not been previously assessed.

Our results demonstrate a substantial increase in pyrethroid-resistant allele frequencies in *Ae. aegypti* from Posadas 5 years after the first local survey in 2019 (27). High kdr allelic frequencies are widely interpreted as evidence of strong selective pressure (11, 29, 31, 35, 36). Long-term monitoring in Brazil and Peru indicated that these alleles may rapidly spread under sustained pyrethroid exposure (32, 37). Kaur et al. (38) recently associated the high prevalence of kdr 1534C (89%) to persistent pyrethroid selection. Locally, vector control protocols in response to dengue outbreaks involve spraying permethrin (*(1-RS)-cis-trans permethrin*) as adulticidal within household premises and by ultra-low volume (ULV) thermal fogging in surrounding public areas (space spraying). These chemical control operations have been performed in the north of Argentina since 1998, with systematic actions since 2003 in Misiones and more intensively during major local dengue outbreaks. This effective containment intervention could, in the long run, increase genetic resistance to insecticides eventually leading to a loss of effectiveness.

Insecticides resistance levels increase with the total number of kdr alleles in each individual (e.g., SS/RR < SR/RR < RR/RR), which is further influenced by synergistic interactions (26). A key outcome of the observed frequency shift is the sharp rise in the number of highly resistant mosquitoes carrying three or more kdr alleles.

We documented a substantial growth (25.56%) in the prevalence of comparatively high resistant mosquitoes between 2019 and 2024, i.e., VI/CC genotypes doubled from 21.05 to 43.41%. Conversely, genotypes with predominant susceptible alleles (VV/FF, VV/FC) have drastically declined to 0.77 and 6.20% for V1016I and F1534C, respectively. If pyrethroid selective pressure persists and kdr allelic frequencies increase at an annual rate of ~5%, the population is likely to become predominantly genotypically resistant.

These findings underscore the importance of routine resistance monitoring as an essential component of vector control programmes and highlight the urgent need to develop and implement alternatives strategies to reduce *Ae. aegypti* population. Although our sample size increased compared with 2019, spatial variation in larvae abundance and in dengue incidence may influence genotype distributions, requiring additional studies completely covering de city. We acknowledge that bioassays offer valuable confirmation of phenotypic resistance; nonetheless, kdr genotyping remains a robust, rapid and cost-effective tool for pyrethroid resistance identification and for temporal trends studies. Another kdr mutation, originally described in Brazil increasing resistance to type I and type II pyrethroids, namely V410L, was recently reported in Argentina contributing to the triple mutant genotype at p.410/p.1016/p.1534 (LL + II + CC) (30, 39). In this work we have not assayed V410L, however, considering the intense LD between p.410 and p.1016, the information collected by genotyping of V410L could be partially inferred by the p.1016 genotyping data readily available (10). In future studies, it would nevertheless be interesting to confirm the co-occurrence of V410L, V1016I and F1534C and to investigate metabolic resistance mechanisms in Posadas *Ae. aegypti* populations. The 5-year comparison presented here provides critical evidence for public health decision-making regarding vector control strategies.

## Data Availability

The raw data supporting the conclusions of this article will be made available by the authors, without undue reservation.
